# Hospital Meetings

**Published:** 1904-06-11

**Authors:** 


### HOSPITAL MEETINGS.

### NORTH-EASTERN HOSPITAL FOR CHILDREN.

The annual meeting of the Governors of this hospital was held on Tuesday, May 31st, Mr. Chas. G. J. Port being in the chair.

The adoption of the report was moved by the Chairman, who said that the expenditure of the hospital had increased very largely, but that had been expected would take place now that they had double the number of beds, and all the work was being done on a better scale than it had ever been before. He wished to call special attention to the financial position of the hospital, which could only be described as being unsatisfactory. They had not received the increased support they had expected from the public and it behoved all supporters of the institution to place the matter before their friends and do what they could to remedy this very serious state of things. The people of the neighbourhood, poor though they were, supported the hospital very well, and if the dwellers at the West End behaved with like generosity the hospital would be in a flourishing condition. The expenditure for building, etc., was nearly £45,000, while the receipts were only £35,000, leaving a debt of over £9,000. This was not so serious as the growing difference between the annual income and expenditure; this last item had gone up, whilst the income had remained nearly stationary.

He had pleasure in informing them that Lord Amherst of Hackney had consented to become president of the hospital.

They hoped that the ceremony of opening the new wards would take place on July 13th, when Princess Louise had graciously promised to declare them open.

This resolution was seconded by Mr. Walter Johnson, who pointed out that the need for the increased accommodation in the hospital was evidenced by the new beds being filled up immediately they were ready for use. The increased expenditure that they would have to face in the future was causing the Committee grave anxiety, and what they wished to impress upon the public was the urgent need of more annual subscribers. It was not as widely known as it should be, that the North-Eastern Hospital was the second largest children's hospital in the metropolis.

The customary votes of thanks to the staff and the re-election of the officials and committee terminated the proceedings.

